# *Hypericum perforatum* L. Nanoemulsion Mitigates Cisplatin-Induced Chemobrain via Reducing Neurobehavioral Alterations, Oxidative Stress, Neuroinflammation, and Apoptosis in Adult Rats

**DOI:** 10.3390/toxics11020159

**Published:** 2023-02-08

**Authors:** Heba M. A. Khalil, Hanan M. A. El Henafy, Islam A. Khalil, Alaa F. Bakr, Mohamed I. Fahmy, Nancy S. Younis, Riham A. El-Shiekh

**Affiliations:** 1 Department of Veterinary Hygiene and Management, Faculty of Veterinary Medicine, Cairo University, Giza 12211, Egypt; 2 Medical Laboratory Department, Faculty of Applied Medical Sciences, October 6 University, Giza 3230911, Egypt; 3 Department of Pharmaceutics, College of Pharmaceutical Sciences and Drug Manufacturing, Misr University of Science and Technology (MUST), Giza 12582, Egypt; 4 Department of Pathology, Faculty of Veterinary Medicine, Cairo University, Giza 12211, Egypt; 5 Department of Pharmacology and Toxicology, Faculty of Pharmacy, Heliopolis University, Cairo 2834, Egypt; 6 Department of Pharmaceutical Sciences, College of Clinical Pharmacy, King Faisal University, Al-Ahsa 31982, Saudi Arabia; 7 Department of Pharmacognosy, Faculty of Pharmacy, Cairo University, Kasr el Aini St., Cairo 11562, Egypt

**Keywords:** cisplatin, *Hypericum perforatum* L., neuroprotective, oxidative stress, inflammation, apoptosis, neurobehavior, toxicity

## Abstract

Cisplatin (Cis) is a potent chemotherapeutic agent; however, it is linked with oxidative stress, inflammation, and apoptosis, which may harmfully affect the brain. *Hypericum perforatum* L. (HP L.) is a strong medicinal plant, but its hydrophobic polyphenolic compounds limit its activity. Therefore, our study aimed to investigate the neuroprotective action of HP L. and its nanoemulsion (NE) against Cis-induced neurotoxicity. The prepared HP.NE was subjected to characterization. The droplet size distribution, surface charge, and morphology were evaluated. In addition, an in vitro dissolution study was conducted. Compared to Cis-intoxicated rats, HP L. and HP.NE-treated rats displayed improved motor activity and spatial working memory. They also showed an increase in their antioxidant defense system and a reduction in the levels of pro-inflammatory cytokines in the brain. Moreover, they showed an increase in the expression levels of the PON-3 and GPX genes, which are associated with a reduction in the brain levels of COX-2 and TP-53. These findings were confirmed by reducing the immunohistochemical expression of nuclear factor kappa (NF-ƘB) and enhanced Ki-67 levels. In conclusion, HP L. is a promising herb and could be used as an adjuvant candidate to ameliorate chemotherapeutic-induced neurotoxicity. Moreover, HP.NE has superior activity in lessening Cis-induced oxidative stress, inflammation, and apoptosis in brain tissue.

## 1. Introduction

Cisplatin (cis-diamminedichloroplatinum II) (Cis) is a universal anticancer agent with recognized neurotoxicity, nephrotoxicity, and hepatotoxicity [1,2]. It is a heavy metal containing a platinum atom surrounded by two chloride molecules and two ammonia molecules [3]. Plasma proteins, including albumin, gammaglobulin, and transferrin, are irreversibly linked to the platinum component of Cis [4]. Due to the prevalence of solid tumor treatment, Cis is one of the most extensively used and recommended anticancer drug [1]. Chemobrain or cognitive impairment is a typical adverse effect of chemotherapeutic medications regularly described by patients treated for cancers [5].

One of the most important mechanisms of Cis neurotoxicity is its ability to induce oxidative stress. Under normal conditions, cells regulate ROS by balancing their generation with their removal by the scavenging mechanism. Extreme damage to cellular proteins, lipids, and DNA is caused by oxidative stress, resulting in significant cellular damage. Cis can induce mitochondrial oxidative damage by disrupting the sulfhydryl group of mitochondrial proteins, thereby impeding calcium uptake and weakening the mitochondrial membrane potential [6], lowering the antioxidant defense system, and decreasing glutathione (GSH) [7]. Programmed cell death in the nervous system is believed to be the primary pathway through which Cis induces neurotoxicity [8].

Since oxidative damage is regarded as a fundamental mechanism of neurotoxicity, using antioxidants to inhibit ROS production is essential for mitigating the effects of such a hazard. The neuroprotective potential of medicinal plants against factor-induced neurotoxicity is still a topic requiring substantial scientific investigation. Both the scientific and consumer groups have accorded *Hypericum perforatum* L. (HP L.), or St. John’s Wort, considerable attention due to its efficacy against various health conditions, including neurological ailments [9]. HP L. is a herbaceous perennial plant native to Europe and Asia and belongs to the Hypericaceae family [10]. It possesses various biological capabilities, including antidepressant, antioxidant, antibacterial, antiviral, and antiproliferative activities [11]. Several phytochemical compounds have been identified in HP L. extract, including naphthodianthrones (hypericin, pseudohypericin, protohypericin, proto-pseudo hypericin, and cyclo-pseudo hypericin), flavonoids, and flavonoid derivatives [11].

Nanoemulsion (NE) was commonly used for improving plant extract pharmacological activities [12,13]. NE’s important advantages are enhancing the solubility of hydrophobic polyphenolic components of extracts and improving the dissolution profile. The simplicity of NE preparation mainly depends on the selection of the NE composition that plays an important role in stability and pharmacological activity. Therefore, several studies have investigated the combination of surfactant, cosurfactant, and oil used to detect the NE region. For example, Li et al. developed NE with Labrafil M 1944 CS (oil), Kolliphor (surfactant), and Transcutol (cosurfactant), and the selected formula was loaded with Lornoxicam [14]. Another study prepared acetazolamide NE for ocular delivery using peanut oil, tween 80/cremophor EL (surfactant), and transcutol/propylene glycol (cosurfactant) [15]. Finally, HP L. was successfully loaded in self-NE drug delivery using triacetin (oil), Tween 20 (surfactant), and PEG 400 (cosurfactant) for post-myocardial infarction depression [16]. 

According to previous research, the phytochemical substance that can lower oxidative stress also has considerable memory-enhancing effects [17]. Although there are numerous studies on the antioxidant effects of HP L. in other organs, less research has been conducted on the neuroprotective effects of HP.NE. Therefore, our research was conducted to evaluate the neuroprotective effect of HP L. and its nano-form (HP.NE) against Cis-induced neurotoxicity. This was accomplished by assessing the neurobehavioral profile of the rats as well as biochemical alterations, including oxidative stress, inflammation, and apoptosis, and gene expression of some regulating genes, including COX-2, TP-53, PON-3, and GPX. Additionally, histopathological changes as well as the immunohistochemical expression of NF-ƘB and Ki-67 were investigated.

## 2. Materials and Methods

### 2.1. Plant Material and Preparation of HP L. Ethanolic Extract

HP L. aerial parts were used in our study, and were purchased from Haraz herbal store, Cairo, Egypt, in 2019 and authenticated by Dr. Mohamed El-Gibali, the Senior Botanist at El-Orman Botanical Garden. A voucher specimen was deposited in the Herbarium of the Pharmacognosy Department, Faculty of Pharmacy, Cairo University (No. 2019-7-18). One kilogram of the powdered plant material was exhaustively extracted with 70% ethanol (5 × 7.5 L) using an Ultra-Turrax^®^ T25 homogenizer (Janke & Kunkel IKA-Lab., Staufen, Germany). The ethanolic extract was evaporated under reduced pressure to dryness (65 g) and then kept in the desiccator over anhydrous CaCl_2_ for biological assay.

### 2.2. Development of Nanoemulsion (NE)

#### 2.2.1. Construction of Pseudo-Ternary Phase Diagram

Pseudo-ternary phase diagrams were constructed to identify the NE region using the titration method. A mixture of surfactant (Tween 80) and co-surfactant (Transcutol HP) (Gattefosse, Lyon, France) was prepared at a 2:1 ratio (SCoSmix). Labrafac (Oil) (Gattefosse, France) and mixed with surfactant/co-surfactant in different volume ratios (from 1:9 to 9:1) in a transparent container. For each Oil/SCoSmix mixture, water was added slowly using the titration method under continuous stirring at room temperature until turbidity appeared [14,18]. The points up to the clarity transition occurred were defined as the phase boundaries. Phase diagrams were drawn using Sigma Plot^®^ software (version 14, Systat Software Inc., San Jose, CA, USA).

#### 2.2.2. Selection of Formulations and Stability Tests

Different formulas were chosen from the NE region as shown in Table 1. Selected formulations were subjected to different stability tests, namely a centrifugation test and a dilution test. For the centrifugation test, the selected formulations were centrifuged at 3500 rpm for 30 min to test creaming or phase separation by visual observation. For the dilution test: the selected formulations were diluted with distilled water 100 times and then visually checked for phase separation and clarity.

#### 2.2.3. Preparation of HP. Loaded NE (HP.NE)

The HP.NE was prepared by a spontaneous emulsification method, as shown in Table 1. HP L. extract (1.5% *w*/*v*) was mixed with oil, then surfactant and cosurfactant were added and mixed until a homogenous mixture was obtained. Water was added gradually under continuous stirring at room temperature. The prepared HP.NE was subjected to characterization. The droplet size distribution and surface charge were measured by Nano-ZS, Malvern Instruments, Malvern, UK. The morphology of the selected formula was visualized by transmission electron microscopy (TEM) (Joel, JEM-2100, Tokyo, Japan).

#### 2.2.4. In Vitro *Dissolution Study*

The in vitro dissolution study was conducted using the dialysis method. About 5 mL of HP.NE was placed into a dialysis bag, placed in 900 mL water (USP II dissolution apparatus, Hanson, MA, USA), and maintained at 37 ± 0.5 °C and 75 rpm. A 5 mL aliquot was withdrawn at predetermined time intervals of 15, 30, 60, 120, and 240 min and replaced by the same volume of water. The HP L. released was measured by a UV/Visible spectrophotometer (Schimadzu, 1600, Kyoto, Japan) with 260 nm as the wave length for the total phenolic compounds, and was then compared with the extract solution.

### 2.3. In Vivo Animal Study

#### 2.3.1. Animals

The current study was carried out with 28 male albino Wistar rats weighing about 170–200 g. The rats were housed in a plastic cage under standard temperature (25 ± 2 °C), humidity (50 ± 5%), and 12:12 h light–dark (LD) cycles. They were provided with a commercial standard diet and water ad libitum.

#### 2.3.2. Experimental Protocol

A total of 28 male rats were randomly distributed into 4 groups, with 7 animals in each, and all groups of rats were treated in the following manner: Group I—Saline/Normal control rats were administered with normal saline (2 mL/kg, p.o. per day) for 14 days and then injected with saline i.p on day 14, followed by oral gavage of saline for another 7 days. Group II—Cis rats were administered with normal saline (2 mL/kg, p.o. per day) for 14 days and injected with Cis (10 mg/kg; Sigma-Aldrich, Inc., St. Louis, MO, USA) i.p on day 14, followed by oral gavage of saline for another 7 days. Group III—*Hypericum* (HP 100 mg/kg + Cis) rats were treated with *Hypericum perforatum* L. (100 mg/kg, p.o. per day) for 14 days and injected with Cis (10 mg/kg) i.p on day 14, followed by oral gavage of HP L. (100 mg/kg) for another 7 days. Group IV—*Hypericum perforatum* L. loaded NE (HP.NE 100 mg/kg + Cis) rats were treated with HP.NE (100 mg/kg, p.o. per day) for 14 days and injected with Cis (10 mg/kg) i.p on day 14, followed by oral gavage of HP.NE (100 mg/kg) for another 7 days. Twenty-four h after the last dose, all groups were subjected to behavioral assessment at 10 a.m., and this continued for 3 days. The dose of Cis was selected according to previous studies [19,20], while the HP L. dose was selected based on our previous research [16].

#### 2.3.3. Neurobehavioral Assessments

Rats were submitted for neurobehavioral assessments, including psychomotor activity using the open field test and spatial working memory using a Y-maze test. In the open field test, rats were placed in the corner of a square wooden arena (70 cm × 70 cm) and allowed to move freely for 3 min. The numbers of crossing squares and rearing frequencies were the measuring parameters [21,22]. In the Y-maze test, each rat was allowed to explore a Y-shaped wooden maze for 5 min. The measuring parameters were the number of arm entries and spontaneous alternation percentage (SAP), as previously reported [22]. All the test devices were cleaned and touched with 70% alcohol to remove any olfactory cues. The assessments were performed by an experienced observer blinded to the experimental groups.

#### 2.3.4. Euthanasia and Brain Sampling

After the last behavioral test, rats were euthanized by cervical dislocation. Brains were excised gently, and half of the brains were rinsed with physiological buffer saline (100 mM Na2HPO4/NaH2PO4, 0.16 mg/mL heparin, pH 7.4) to remove RBCs and clot residues. Then, 1 g of brain sample was homogenized in 5 mL of cold phosphate-buffered saline (50 mM potassium phosphate, 1 mM of ethylenediaminetetraacetic acid [EDTA], pH 7.5) using a sonic homogenizer. All set homogenates were centrifuged at 14,000× *g* for 15 min at 4 °C. The supernatant was used to measure the oxidative biomarkers and proinflammatory cytokines. The other halves of the brains were preserved in neutral buffer formalin 10% for subsequent histopathological investigation.

#### 2.3.5. Biochemical Assay

##### Oxidative Biomarkers Assay

The antioxidant enzymatic activities of superoxide dismutase (SOD), glutathione -s- transferase (GST), and catalase (CAT), with the level of reduced glutathione (GSH), and the lipid peroxidation marker, malondialdehyde (MDA), nitric oxide concentration (NO) level were evaluated in the supernatant as previously mentioned according to the manufacturer’s guide notes (Laboratory Biodiagnostic Co., Giza, Egypt).

##### Evaluation of Pro-Inflammatory Cytokine Levels

The level of interleukin-6 (IL-6), interleukin-1-beta (IL-1β), tumor necrosis factor- α (TNF-α), caspase-3, and 8-hydroxy-20-deoxyguanosine (8-OHdG) were evaluated in the brain supernatant by using ELISA kits from ThermoFisher scientific Invitrogen (Waltham, MA, USA) and Abcam (Cambridge, UK), according to the manufacturer’s manual guidelines.

##### Quantitative of COX-2, TP-53, PON-3, and GPX Gene Expression by qRT-PCR

RNA was purified from 100 mg of rat brain tissue using the RNA purification kit (Thermo Scientific, Waltham, MA, USA, GeneJET #K0734) according to the manufacturer’s protocol. The concentration of the total RNA was assessed spectrophotometrically (Thermo Scientific, USA). The isolated RNA was reverse transcribed into cDNA and used for PCR with specific primers. The sequences of these primers were designed by https://www.ncbi.nlm.nih.gov/tools/primer-blast/ access date 9/2022, and are as follows: COX-2 forward 5′-AAA GCC TCGTCCAGATGC TA-3′, reverse 5′-ATGGTGGCTGTCTTGGTAGG-3′; TP-53 forward 5′-GTG GTA CCG TAT GAG CCA CC-3′, reverse 5′-CAA CCT GGC ACA CAG CTT CC-3′; PON-3 forward5′-CATCCAGGATCCTTTGTCAGATAA3′, reverse 5′ CACGGTGCTGCCCTGAAG-3′; GPX forward 5′ CGGTTTCCCGTGCAATCAGT3′, reverse 5′- ACACCGGGGACCAAATGATG-3′; and β-Actin (housekeeping gene) forward 5′ CACCCGCGAGTACAACCTTC 3′, reverse 5′ CCCATACCCACCATCACACC 3′. SYBR Green PCR Master Mix was used to evaluate the studied genes. cDNA was added to an SYBR Green qPCR Master Mix (Thermo Scientific, USA) containing 30 pg/mL of each primer. The product was amplified by 40 cycles of denaturation at 95 °C for 15 s, annealing at 60 °C for 15 s, and extension at 72 °C for 45 s. During the first cycle, the 95 °C steps were extended to 1 min. The β-Actin gene was amplified in the same reaction to serve as the reference gene. Every sample was analyzed in duplicate. The gene expression level was calculated using the comparative cycle threshold method [23].

#### 2.3.6. Histopathological Analysis

Sagittal sections from brain tissue were fixed in paraformaldehyde (4%) and embedded in paraffin blocks. After that, blocks were cut into thin slices (5–6 μm) and stained with hematoxylin and eosin staining [24]. Different brain regions were examined in each animal by using an Olympus light microscope (BX43, USA) connected to a DP27 digital camera and CellSens dimensions software. The histological damage score was assessed in each animal according to previous studies [25,26]. Briefly, variant lesions, including neuronal degeneration, neurophagia, gliosis, and necrosis, were scored in different brain regions according to the severity as follows: 0 = absent, 1 = sporadic neuron (<25%), 2 = distributed neurons (25–50%), 3 = distributed neurons (>75), 4 = multifocal (>75). The final score for each animal was obtained by summation of the total score for multiple lesions (4–5 fields/200×).

#### 2.3.7. Immunohistochemistry (IHC) Analysis for Inflammation and Cell Proliferation Biomarkers

Deparaffinized brain tissues were rehydrated and submitted to peroxidase-blocking procedures. Subsequently, slides were incubated with the primary antibody of NF-ƘB or Ki-67 (1:100). Later, the slides were rinsed with PBS three times and incubated with the secondary antibody. Color enrichment was carried out by diaminobenzidine (2 mL, 15 min), and hematoxylin was used as counterstain. Positive results were obtained as area % using Image J software version 1.47 (http://imagej.en.softonic.com/, accessed on 3 February 2023).

### 2.4. Statistical Analysis

Neurobehavioral parameters and biochemical data were expressed as mean ± standard error (SE), while histopathological scoring was expressed as median ± standard deviation. The statistical analyses were performed using the one-way analysis of variance (ANOVA) followed by Tukey’s post hoc test or the Kruskal–Wallis test, followed by Dunn’s multiple comparison tests for histopathological brain scoring. Histograms were plotted using Graph Pad Prism 9.4.1 (Graphpad Software, San Diego, CA, USA). Differences were accepted when *p* was <0.05, <0.01, or <0.001.

## 3. Results

### 3.1. Development of HP. NE

**Figure 1:**
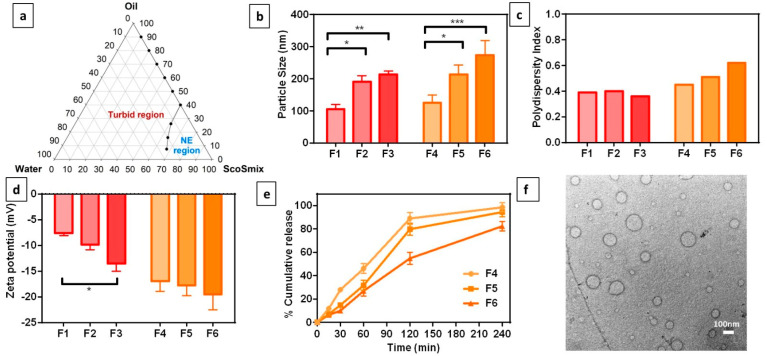
Development of *Hypericum perforatum* L. nanoemulsion (HP.NE): (**a**) Ternary phase diagram represents the prepared 9 screening trials where the borderline separates the NE region. (**b**) Globule size, (**c**) polydispersity index, and (**d**) surface charge of unloaded (F1–3) and loaded (F4–6) selected NEs. (**e**) The in-vitro release profile of loaded NEs. (**f**) TEM image of F4 as the selected formulation for further investigations. * means significant at *p* < 0.05; **, significant at *p* < 0.01; ***, significant at *p* < 0.001).

The pseudo-ternary phase diagram was constructed to identify the NE region, where 9 trials were investigated using oil and SCoSmix in different volume ratios (from 1:9 to 9:1). According to Figure 1a, only three trials showed a clear transparent NE with low oil percentage and high SCoSmix percentage. Oil percentage (7.5% to 26.1%), and SCoSmix (60.9% to 67.5%) were used to draw the borderline for the NE region (Figure 1a). Hence, three formulations in the middle of NE were selected for further investigations. The first step was testing formulation stability by centrifugation test using visual observation of creaming or phase separation along with a dilution test to visually check for phase separation and clarity. As shown in Table 1, F1–F3 formulations were visually clear after both centrifugation and dilution tests. Therefore, HP L. at concentration 1.5% *w*/*v* was incorporated in the NE (F4–F6). The globule size of the unloaded NE ranged from 105.2 ± 10 to 213.7 ± 11 nm (Figure 1b), with PDI ranging from 0.36–0.4 (Figure 1c). The surface charge ranged from −7.61 to −13.54 mV (Figure 1d). Loading HP L. in NE showed slight increases in the size, PDI, and surface charge. The globule size of F4–F6 was 125.2 ± 24 to 273.7 ± 45 nm (Figure 1b), with PDI ranging from 0.45–0.61 (Figure 1c). The surface charge ranged from −16.94 to −19.49 mV due to the presence of polyphenolic compounds (Figure 1d). The dissolution profile of HP.NE (F4–F6) was determined using the dialysis technique (Figure 1e). The dissolution profile of F4 with 10% oil and 80% SCoSmix showed the fastest dissolution, with around 50% and 100% released after 1 h and 4 h, respectively. Decreasing SCoSmix to 70% slightly slowed the release, as shown for F5. The slowest formula was F6 with 20% oil and 70% SCoSmix, where around 25% and 80% were released after 1 h and 4 h, respectively. Therefore, F4 with 10% oil and 80% SCoSmix was selected for further investigation. A TEM image of F4, represented in Figure 1f, shows spherical NE globules with sizes near to the size measured by the dynamic light scattering technique.

### 3.2. Effect of HP L. and HP.NE Administration on the Neurobehavioral Parameters of the Cis-Intoxicated Rats

**Figure 2:**
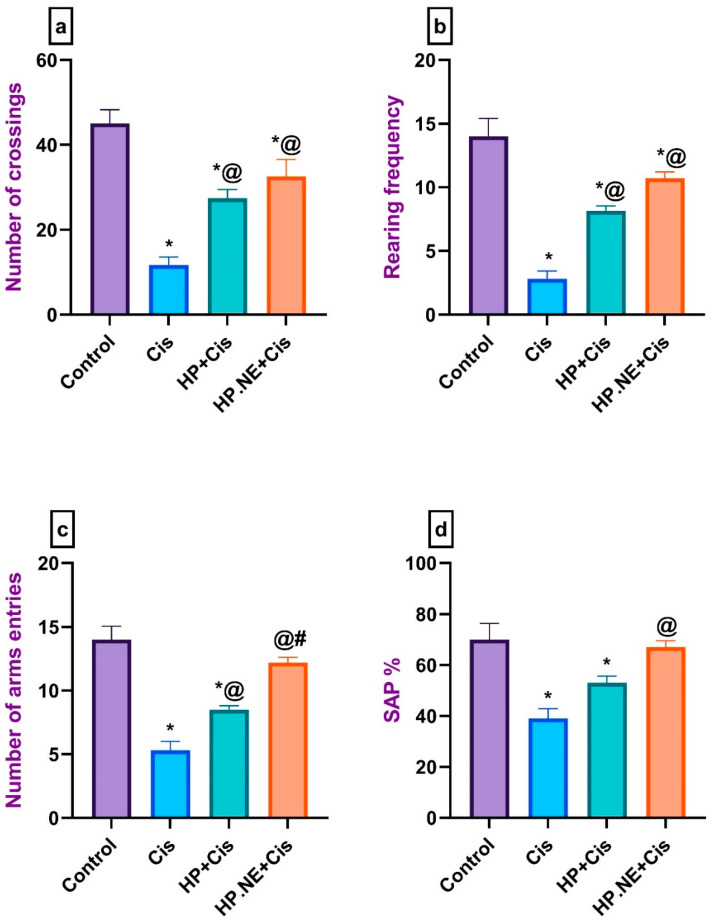
Effect of HP L. and HP.NE administration on the motor activity and spatial working memory of Cis-intoxicated rats. (**a**) Open field test: number of crossing squares, (**b**) open field test: rearing activity, (**c**) Y-maze test: number of arm entries, and (**d**) Y-maze test: spontaneous alternation percentage (SAP). Data are expressed as mean ± standard error (SE)using one-way analysis of variance (one-way ANOVA) followed by Tukey’s post hoc test for seven rats in each group. * means significant from the Control group; @, significant from the Cis group; #, significant from the HP + Cis group.

As displayed in Figure 2, Cis-intoxicated rats displayed a significant reduction in their motor activities, evidenced by a decrease in the number of crossing squares and the rearing frequency in the open field test and a marked reduction in the number of arm entries in the Y-maze test compared to control rats. However, administration of HP L. and its formulation, HP.NE, succeeded in improving the general locomotion of rats compared to Cis-intoxicated rats (Figure 2a–c). Concerning SAP in the Y-maze test, Cis-intoxicated rats exhibited a marked reduction in their SAP compared to control rats. On the other hand, HP L. displayed a marked increase in the SAP compared to Cis-intoxicated rats (Figure 2d). Remarkably, HP.NE-treated rats displayed a noticeable improvement in all the aforementioned parameters over HP-treated rats.

### 3.3. Effect of HP L. and HP.NE Administration on the Levels of Oxidative Biomarkers of the Cis-Intoxicated Rats

**Figure 3:**
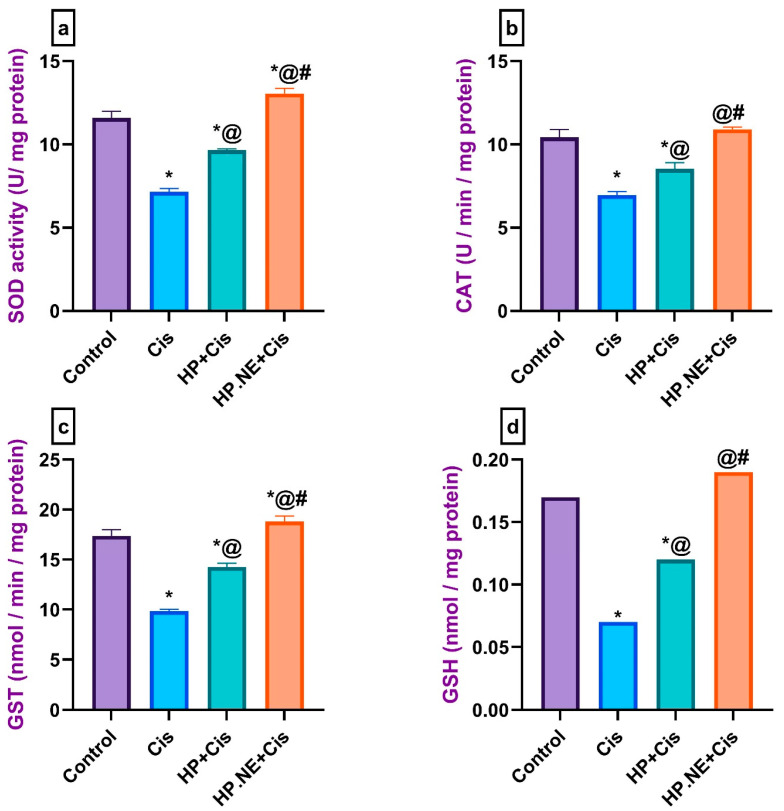
Effect of HP L. and HP.NE administration on the brain levels of oxidative biomarkers of different groups. (**a**) SOD: super oxide dismutase, (**b**) CAT: catalase, (**c**) GST: glutathione -s- transferase, and (**d**) GSH: reduced glutathione. Data are expressed as mean ± SE (one-way ANOVA) followed by Tukey’s post hoc test for seven rats in each group. * means significant from the Control group; @, significant from the Cis group, #, significant from the HP + Cis group.

As shown in Figure 3, SOD, CAT, GST, and GSH brain levels were significantly decreased in the brains of Cis-intoxicated rats compared to control rats. In contrast, HP L. and HP.NE-treated rats exhibited a marked increase in the brain levels of SOD, CAT, GST, and GSH compared to Cis-intoxicated rats.

**Figure 4:**
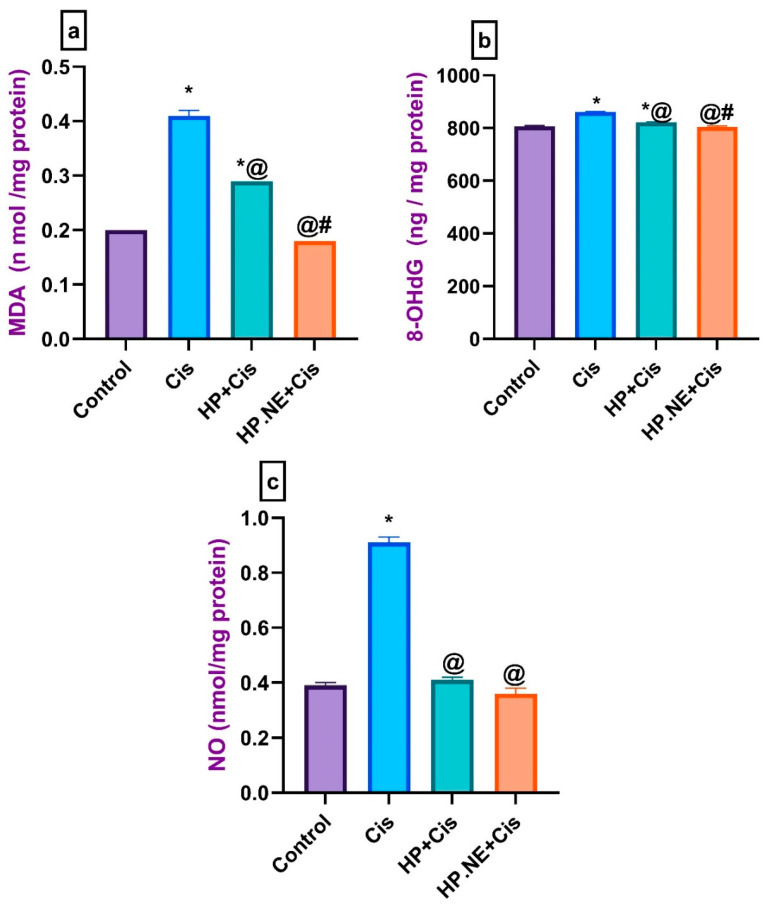
Effect of HP L. and HP.NE administration on the brain levels of malondialdehyde, 8-hydroxy-20-deoxyguanosine, and nitric oxide for different groups. (**a**) MDA: malondialdehyde, (**b**) 8-OHdG: 8-hydroxy-20-deoxyguanosine, and (**c**) NO: nitric oxide. Data are expressed as mean ± SE (one-way ANOVA) followed by Tukey’s post hoc test for seven rats in each group. * means significant from the Control group; @, significant from the Cis group; #, significant from the HP + Cis group.

Conversely, Cis-intoxicated rats showed a substantial increase in the brain levels of MDA, 8-OHdG, and NO compared to the control group. However, HP L. and HP.NE-treated rats exhibited a significant decrease in these levels compared to Cis-intoxicated rats (Figure 4). Furthermore, HP.NE was found to be more effective in improving antioxidant mechanism enzymes as compared to the HP-treated rats.

### 3.4. Effect of HP L. and HP.NE Administration on the Levels of Pro-Inflammatory Cytokines in Cis-Intoxicated Rats

**Figure 5:**
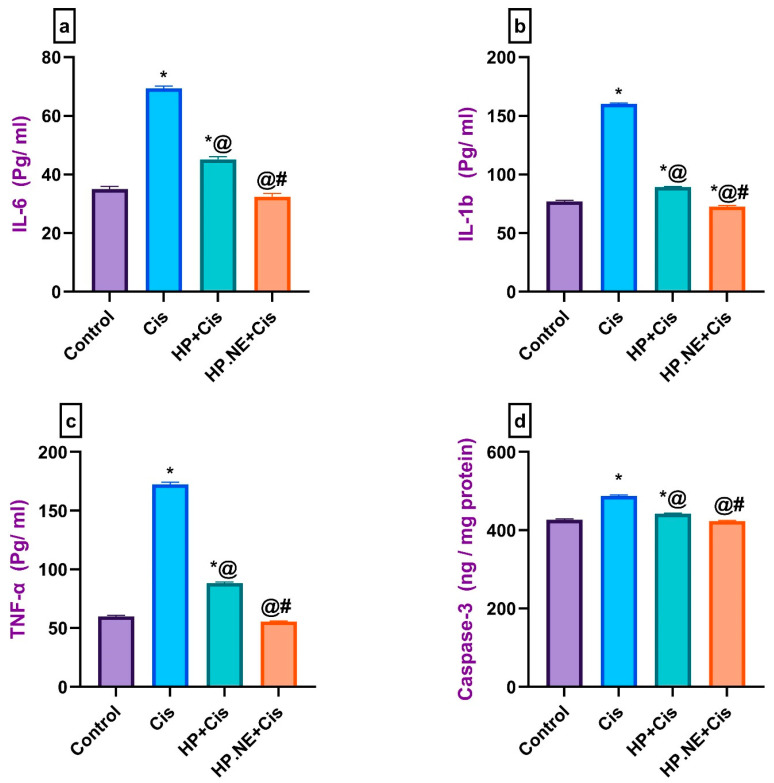
Effect of HP L. and HP.NE administration on the brain levels of pro-inflammatory cytokines in different groups. (**a**) IL-6: interleukin 6, (**b**) IL1b: interleukin-1 beta, (**c**) TNFα: tumor necrosis factor α, and (**d**) Caspase-3. Data are expressed as mean ± SE (one-way ANOVA) followed by Tukey’s post hoc test for seven rats in each group. * means significant from the Control group; @, significant from the Cis group; #, significant from the HP + Cis group.

As depicted in Figure 5, Cis-intoxicated rats’ brains displayed a marked neuroinflammation, evidenced by a substantial increase in the brain levels of IL-6, IL-1β, TNF-α, and caspase-3 compared to control rats. Treatment with HP L. and HP.NE exerted an anti-inflammatory action evidenced by decreasing the aforementioned pro-inflammatory cytokines compared to Cis-intoxicated rats. Interestingly, HP.NE administration was found to be more effective in modulating neuro-inflammation, as shown by a significant reduction in the brain levels of these pro-inflammatory cytokines.

### 3.5. Effect of HP L. and HP.NE Administration on the Expression Levels of PON-3 and GPX Genes in Cis-Intoxicated Rats

**Figure 6:**
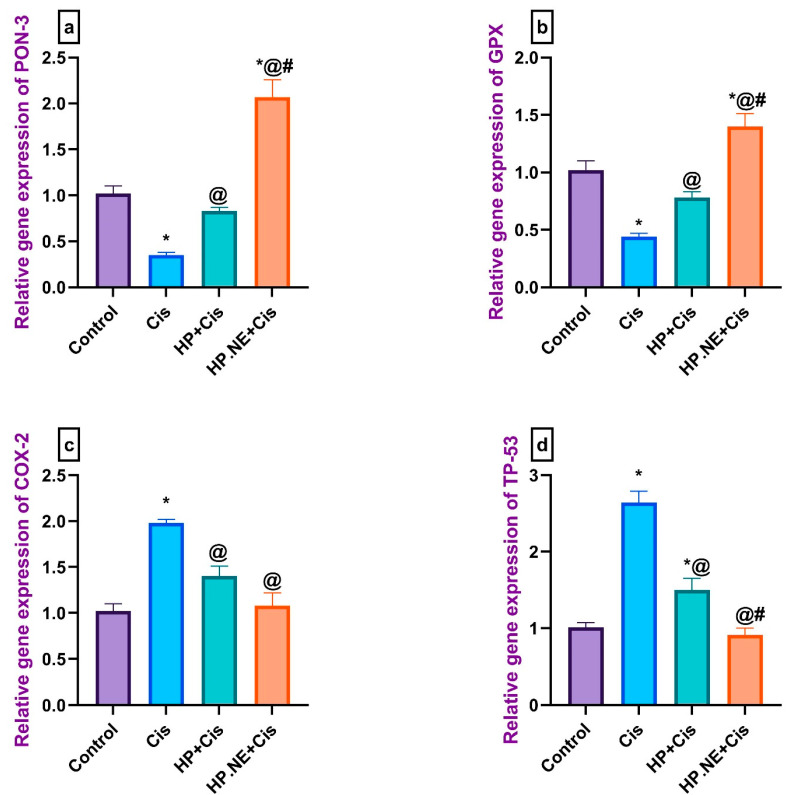
Effect of HP L. and HP.NE administration on the relative gene expression of PON-3, GPX COX-2, and TP-53 in brain tissues of different groups. (**a**) PON-3: paraoxonase 3, (**b**) GPX: glutathione peroxidase, (**c**) COX-2: cyclooxygenase-2, and (**d**) TP-53: tumor protein p53. Data are expressed as mean ± SE (one-way ANOVA) followed by Tukey’s post hoc test for seven rats in each group. * means significant from the Control group; @, significant from the Cis group; #, significant from the HP + Cis group.

Cis-intoxicated rats displayed a substantial downgrade of the expression levels of the PON-3 and GPX genes compared to the control rats. Meanwhile, HP L. and HP.NE-treated rats displayed a marked upgrade in the expression levels of these genes compared to the Cis-intoxicated rats. Likewise, the administration of HP.NE was found to be more effective in increasing the expression levels of PON-3 and GPX genes in the brain than in the HP-treated rats (Figure 6a,b).

### 3.6. Effect of HP L. and HP.NE Administration on the Expression Levels of COX-2 and TP53 Genes in Cis-Intoxicated Rats

Cis-intoxicated rats displayed a significant increase in the expression levels of COX-2 and TP53 in the brain compared to the control rats. Meanwhile, HP L. and HP.NE-treated rats displayed a marked reduction in the expression levels of these apoptotic genes compared to the Cis-intoxicated rats. Moreover, the administration of HP.NE was found to be more effective in decreasing the expression levels of TP53 and COX-2 genes in the brain compared to the HP-treated rats (Figure 6c,d).

### 3.7. Histopathological Investigation

**Figure 7:**
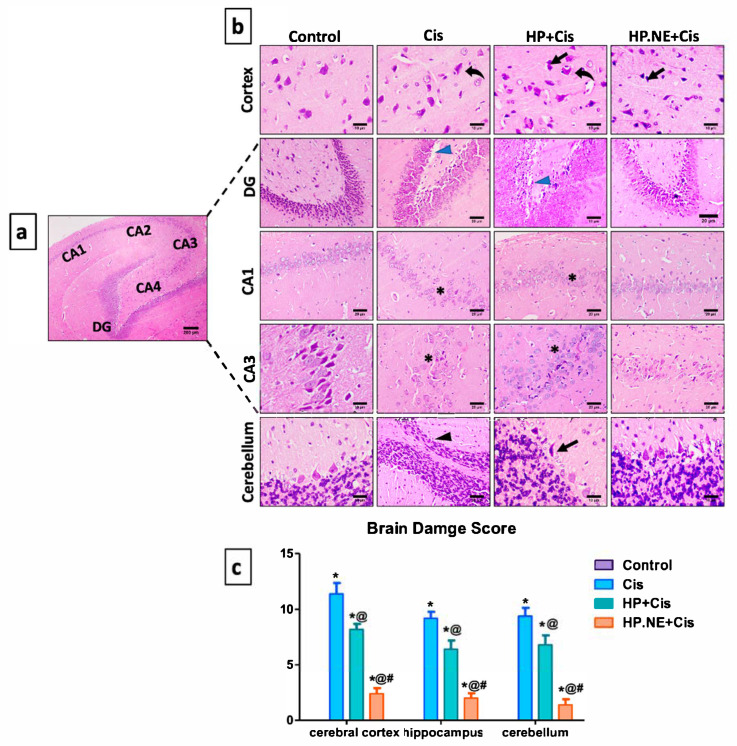
Effect of HP L. and HP.NE administration on the histological structure of the brain. (**a**) Photomicrograph from control group presenting the normal structure of the hippocampal area, which is made up of Cornu Ammonis (CA1, CA2, CA3, and CA4) and Dentate gyrus (DG); scale bar denotes 200 m. (**b**) Photomicrograph of brain tissues stained with H&E; scale bars denote 10 and 20 m. Notable symbols on the figure point to neurophagia of degenerated neurons (curved arrows), dark stained neurons (arrows), loss of neurons (black arrowheads), areas void of neurons (*), and vacuolation (blue arrowheads). (**c**) Brain damage score. Results are expressed as median standard deviation (SD), determined using the Kruskal–Wallis test followed by Dunn’s multiple comparison test. * means significant from the control group; @, significant from the Cis group; #, significant from HP + Cis group.

In our histological analysis, we focused on three different brain regions: cerebral cortex, hippocampus, and cerebellum. As depicted in Figure 7, the control group revealed normal histological structures of different brain sections. Briefly, the cerebral cortex consists of intact neurons and neuroglia cells. The hippocampus region showed regular construction of the subiculum, dentate gyrus, and cornus ammonis (CA) that split into four areas (CA1–CA4) according to the volume and distribution of pyramidal cells. Further, the cerebellum exhibited a normal molecular layer, a granule cell layer, and a large, active basophilic purkinje cell with a vesicular nucleus.

Cis can induce undesirable changes in the histological structure of brain that present as massive neuronal degeneration with neuronophagia in the cerebral cortex. Regarding the hippocampal area, apoptosis of pyramidal cells, as well as distinct vacuolation, were detected in dentate gyrus. Furthermore, the Cis group revealed significant degeneration and nonappearance of neurons in different arenas of CA1 and CA3. Moreover, degenerated purkinje cells were observed in the cerebellum.

Administration of HP L. and HP.NE to Cis-intoxicated rats improved and maintained the histological structure of the brain tissues against Cis toxicity. However, the greater protective effect against Cis was attributed to HP.NE.

### 3.8. Immunohistochemical Investigation of NF-ƘB and Ki-67 Protein Expression

**Figure 8:**
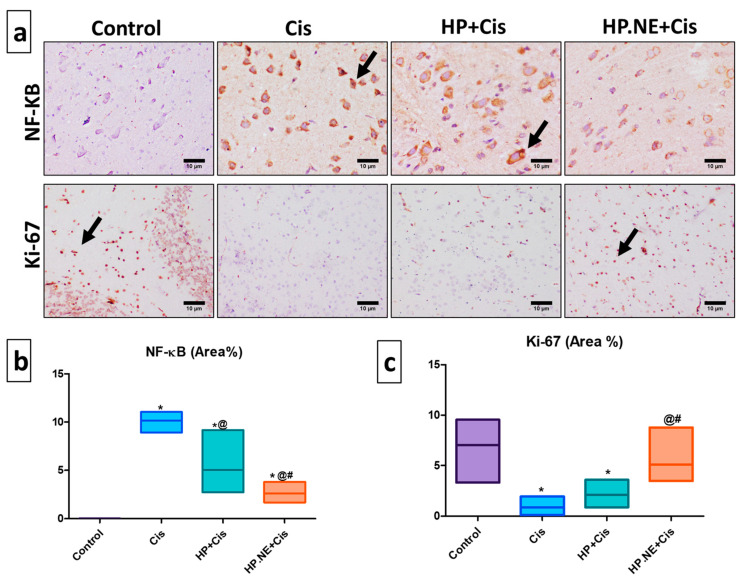
Immunohistochemical staining of inflammatory Nuclear factor kappa B (NF-ƘB) and proliferative (Ki-67) markers in brain tissues of all groups. (**a**) Photomicrograph of brain tissues stained with NF-ƘB and Ki-67 (H&E); scale bars denote 10 µm. Remarkable arrows on the figure point to strong positive staining. (**b**) Statistical analysis of NF-ƘB expression (area%). (**c**) Statistical analysis of Ki-67 expression (area%). * means significant from the control group; @, significant from the Cis group; and #, significant from HP + Cis group.

As depicted in Figure 8, Cis and HP-treated rats revealed strong upregulation of NF-ƘB expression compared to the control group, while HP.NE-treated rats exhibited weak expression compared to the control and HP-treated rats. Remarkably, strong positive brown Ki-67 protein expression was recorded in both the control and HP.NE-treated rats, whereas marked expression reduction was detected in the Cis and HP-treated rats. It is worth mentioning that the expression of KI-67 in the group that received HP.NE was greater than those who received HP L. extract.

## 4. Discussion

In our study, we investigated the neuroprotective effect of HP L. and its nano-form (NE) against Cis-induced chemobrain while addressing the causes underlying this disorder, including locomotion disturbance, spatial working memory alterations, oxidative stress, and apoptosis. In addition, an emphasis on some regulatory genes, including PON-3, GPX, TP53, and COX2, has been reported. The selected HP.NE was composed of 10% Labrafac, 53.3% Tween 80, 26.6% Transcutol, and 10% water, whereas HP L. was 1.5% *w*/*v*. The globule size was 125.2 ± 24 nm, and the surface charge was −16.94 ± 3 mV due to the presence of polyphenolic compounds. A mixture of surfactant (Tween 80) and co-surfactant (Transcutol HP) was used at a 2:1 ratio (SCoSmix). Transcutol HP is usually used at a low ratio when mixed with oil due to its ability to spontaneously develop NE, as previously reported with Tadalafil NE (Oil: Capmul and SCoSmix: Labrasol/Transcutol HP) [18]. For HP encapsulation, a study encapsulated HP (concentration 1.5 to 6 mg/mL) in NE using 8% Lipoid, 2% egg lecithin, 1% tween 80 as SCoSmix, and ethanol +acetone as a solvent mixture. Although this method successfully prepared NE with a size of less than 200 nm, it requires the evaporation step of organic solvent and testing the residual solvent [27]. Another study reported self-NE loaded with HP L. for post-myocardial infarction depression. This study used self-nano-emulsification and in situ nanosuspension as a way to solubilize HP L. using 10% triacetin, 57% Tween 20, and 33% PEG 400 as surfactants and cosurfactants. The size was 258.65 nm, and the charge was −8.03 mV. The HP self-NE released 72.3% of HP L. in 5 min, which indicates a significant improvement in HP L. solubility [16]. These findings are in contrast with our study, where the development of HP.NE controlled the dissolution profile over 4 h. This could be attributed to the differences in the nature of the oil and surfactant system used in the current study.

The exposure of rats to Cis (10 mg/kg) caused a marked behavioral alteration, including locomotion disturbance, as portrayed by the decrease the number of crossing squares and the rearing frequency in the open field test, as well as the decrease the number of arm entries in the Y-maze test, compared to the control group. An open field test is an experimental test used to measure the locomotor activity level, anxiety, and exploratory behavior [22]. This test depends on the assumption of the natural fear of rodents to open illuminated areas. The reduction in the number of crossing squares reflects motor deficits and a high state of emotionality, and decreasing the rearing frequency reflects a reduction in exploratory behavior. These results are in agreement with a previous study conducted by Kandeil et al. [28], who investigated the locomotor and memory impairment associated with Cis administration. In addition, Ongnok et al. discussed the pathogenesis of chemotherapeutic medications on the brain in their study [29]. Besides locomotor disturbance, Cis treatment caused impairments in cognitive function and learning abilities [30]. Herein, we used the Y-maze test, a hippocampal-dependent test, to measure the spatial working memory in response to Cis toxicity. Memory impairment was evidenced by a decrease in the percentage of SAP. This parameter depends on the spontaneous ability of rats to alternate between three different arms [31]. Working memory processing depends on the intactness of several brain regions, such as the prefrontal cortex, hippocampus, and striatum [32]. This finding agrees with a previous study by Ongnok et al. [29].

These neurobehavioral alterations were confirmed by examining different brain regions, including the cerebral cortex, hippocampus, and cerebellum. Cis induced several histopathological alterations in the brain tissue [7]. Similarly, in our study, the exposure of rats to Cis induced degeneration and neurophagia in the cerebral cortex, as well as vacuolation and loss of pyramidal neuron in the hippocampal area. Furthermore, deeply stained Purkinje cells were detected in the cerebellum compared to control group. This could be due to the ability of Cis to cross the blood–brain barrier (BBB) and suppress neural stem cell proliferation, causing neurotoxicity [4]. Several pathways for Cis neurotoxicity have been proposed, with oxidative damage being one of the most significant in Cis and other chemotherapeutic medications [33]. Oxidative stress affects cell structure and function while decreasing antioxidant mechanisms and damaging DNA [2]. In this study, our data demonstrated that Cis considerably induces severe cerebral oxidative stress, as indicated by the severe depletion of SOD, GST, CAT, and GSH levels, along with the concurrent augmentation of MDA, 8-OHdG, and NO. These results align with previously reported studies [34,35].

PON-3 and GPX are essential antioxidant genes; their capacity to prevent lipid oxidation makes them necessary for the myelination of nerves in the brain. Their protein products interact with high-density lipoproteins in the blood. Xie et al. demonstrated that oxidative stress induced by Cis is associated with decreased PON-3 and GPX expression in brain tissue [36]. Similarly, the Cis-intoxicated group displayed a marked reduction in PON-3 and GPX expression compared to the control group.

Inflammation is another pathway of Cis-induced neurotoxicity [37]. NF-ƘB is considered the master regulator of inflammation and it can mediate the upregulation of pro-inflammatory cytokines and chemokines [38]. In our study, Cis-intoxicated rats showed an increase in the expression levels of brain NF-ƘB, which stimulates the production of pro-inflammatory cytokines, including IL-6, IL-1β, TNF-α, and caspase-3, compared to control rats. Furthermore, Cis produced a marked increase in the brain levels of COX2 and TP53 compared to the control group. Under normal conditions, the apoptotic gene (COX2) is expressed in the CNS [39]. Additionally, the TP53 gene mediates the regulation of the COX2 gene [40]. The overexpression of COX2 and TP53 are both signs and causes of brain injury [39]. The excessive production of NO as a result of neurotoxicity may be the primary cause of COX2 and TP53 overexpression. Furthermore, Cis induces downregulation in the expression levels of the Ki-67 marker, which is responsible for the ability of brain tissue to survive restoration following injury [41]. This finding is attributed to the oxidative stress and inflammation elicited by Cis that could be consistent with the inhibition of neurogenesis [41].

Natural products are sources of antioxidant and anti-inflammatory components, including phenolic compounds such as chlorogenic acid [42,43,44], caffeoylquinic acid derivatives [45], procyanidins [46], and catechins [47]. Flavonoids are phytochemicals which have attracted considerable attention because of their many pharmacological interests and health-promoting uses. Myricetin and its glycosides, which were previously identified in our extract (Figure S1 and Table S1) [16], have great antioxidant, anti-inflammatory, and antiapoptotic potential [48,49]. Rutin is also the major compound in our extract; it exhibited several pharmacological effects and was reported as a good alternative to conventional drugs for oxidative stress and inflammation [50]. HP L. is a phenolics-enriched extract; thus, it could inhibit the production of ROS and, in consequence, prevent apoptosis. Rutin, myricetin, hyperoside, quercetin, kamepherol, hyperforin, and hypericin are among the identified metabolites in our extract (Figure S1 and Table S1) [16]. The role of polyphenolics as antiapoptotic agents has been reported in many studies [51,52,53]. Incorporation of HP L. into an NE system was found to effectively ameliorate the neurobehavioral alterations caused by Cis, represented by increasing the number of crossing squares and the rearing frequency in the open field test and increasing the number of arm entries and SAP in the Y-maze test. Several studies have investigated the protective benefits of HP L. against cognitive impairment in neurodegenerative disorders [16,54].

Moreover, the antioxidant property of HP L. and HP.NE was evidenced by lowering the brain levels of MDA, 8-OHdG, and NO and increasing in the cerebral antioxidant defense of SOD, GST, CAT, and GSH. The antioxidant activities of HP L. and HP.NE are correlated with their ability to act as free radical scavengers by inhibiting lipid peroxidation. These findings were associated with promoting the expression of PON-3 and GPX antioxidant genes in the brain tissue. As we mentioned before, this could be attributed to the bio-flavonoid content of HP L. and HP.NE, which has a direct scavenging effect on ROS [55].

Furthermore, the anti-inflammatory property of the HP L. and HP.NE was portrayed by decreasing the brain levels of IL-6, IL-1β, TNF-α, and caspase-3. This finding may be explained by the ability of HP L. and HP.NE to scavenge free radicals and inhibit enzyme leakage across plasma membranes [49]. Notably, the pro-inflammatory cytokines in HP.NE treated rats were comparable to those in control rats. In addition, the expression levels of COX-2 and TP-53 were decreased in the HP L. and HP.NE-treated rats. Wu et al. revealed that antioxidants inhibit COX2 expression in brain tissue [56] by inhibiting activator protein1 (AP1), which regulates the promoters of different inflammatory genes (such as COX2 and TP53). Furthermore, Cui et al. showed that antioxidants down-regulate COX2 and TP53 production via inhibiting NF-κ activity in the same sequence [57]. The anti-inflammatory activity of HP L. and HP.NE may be partially attributable to their ability to modulate COX2 and TP53 expression [58]. Alternatively, it has been demonstrated that hyperforin acts as a dual inhibitor of TP53 and COX2 in intact cells and on the catalytic activity of the enzymes, suggesting therapeutic promise for inflammatory diseases [59]. These findings were further confirmed by assessing the expression levels of the Ki-67 marker that was considered a marker for newly formed cells in brain tissue after exposure to damage [60]. Treatment with HP L. and HP.NE promoted proliferation in the brain tissue after Cis administration via significant improvement in the Ki-67 marker. These results were consistent with previous studies that demonstrated the anti-inflammatory and preservative action of HP L. [16,61]. Based on the previously mentioned results, utilizing antioxidant, anti-inflammatory, and anti-apoptotic agents such as HP L. and HP.NE to guard against Cis-induced cerebral apoptosis is a logical strategy.

## 5. Conclusions

In conclusion, the administration of HP L. and HP.NE appeared to be an effective therapy for counteracting the neurobehavioral alterations, brain oxidative stress, inflammation, and apoptosis induced by Cis, as evidenced by the marked improvement in the behavioral profile of treated rats, significant reduction in lipid peroxidation markers, pro-inflammatory cytokines, elevated PON-3 and GPX, and suppression of TP53 and COX2 brain levels. In addition, a reduction in the expression of brain levels of NF-ƘB associated with an increase in the brain levels of Ki-67 was confirmed. Similarly, the brain architecture was restored. These results could be attributed to the phytochemical profile of HP L., which possesses antioxidant, anti-inflammatory, and antiapoptotic compounds. The medicinal activity of HP L. was improved by incorporating HP L. into the NE system, where the nanosized and lipophilic nature of the carrier improved the absorption of the phytochemical components of HP L.

## Figures and Tables

**Table 1:** NE composition and physical stability.

F#	HP L. Extract % *w*/*v*	Oil % *w*/*w*	SCoSmix % *w*/*w*	Water % *w*/*w*	Centrifugation Test	Dilution Test
F1	-	10	80	10	clear	clear
F2	-	10	70	20	clear	clear
F3	-	20	70	10	clear	clear
F4	1.5	10	80	10	-	-
F5	1.5	10	70	20	-	-
F6	1.5	20	70	10	-	-

The symbol “-” and the word “clear” in the table denote zero.

## Data Availability

Data will be available upon request from authors.

## Supplementary Materials

The following supporting information can be downloaded at: https://www.mdpi.com/article/10.3390/toxics11020159/s1, Figure S1: Total ion chromatogram of HP L. ethanolic extract in positive ionization mode and Table S1: Metabolites identified from the ethanolic extract of HP L.

## References

[1] Üstün R., Oğuz E.K., Şeker A., Korkaya H. (2018). Thymoquinone Prevents Cisplatin Neurotoxicity in Primary DRG Neurons. Neurotoxicology.

[2] Hosseinzadeh M., Alizadeh A., Heydari P., Kafami M., Hosseini M., Beheshti F., Marefati N., Ghanbarabadi M. (2021). Effect of Vitamin E on Cisplatin-Induced Memory Impairment in Male Rats. Acta Neuropsychiatr..

[3] Dasari S., Bernard Tchounwou P. (2014). Cisplatin in Cancer Therapy: Molecular Mechanisms of Action. Eur. J. Pharmacol..

[4] Mehmood R.K. (2014). Review of Cisplatin and Oxaliplatin in Current Immunogenic and Monoclonal Antibody Treatments. Oncol. Rev..

[5] O’Farrell E., MacKenzie J., Collins B. (2013). Clearing the Air: A Review of Our Current Understanding of “Chemo Fog”. Curr. Oncol. Rep..

[6] Kleih M., Böpple K., Dong M., Gaißler A., Heine S., Olayioye M.A., Aulitzky W.E., Essmann F. (2019). Direct Impact of Cisplatin on Mitochondria Induces ROS Production that Dictates Cell Fate of Ovarian Cancer Cells. Cell Death Dis..

[7] Arafa M.H., Atteia H.H. (2020). Protective Role of Epigallocatechin Gallate in a Rat Model of Cisplatin-Induced Cerebral Inflammation and Oxidative Damage: Impact of Modulating NF-ΚB and Nrf2. Neurotox. Res..

[8] Gorgun M.F., Zhuo M., Englander E.W. (2017). Cisplatin Toxicity in Dorsal Root Ganglion Neurons Is Relieved by Meclizine via Diminution of Mitochondrial Compromise and Improved Clearance of DNA Damage. Mol. Neurobiol..

[9] Oliveira A.I., Pinho C., Sarmento B., Dias A.C.P. (2016). Neuroprotective Activity of *Hypericum perforatum* and Its Major Components. Front. Plant Sci..

[10] Marrelli M., Statti G., Conforti F., Menichini F. (2016). New Potential Pharmaceutical Applications of Hypericum Species. Mini Rev. Med. Chem..

[11] Prakash D.J., ArulKumar S., Sabesan M. (2010). Effect of Nanohypericum (*Hypericum perforatum* Gold Nanoparticles) Treatment on Restraint Stressinduced Behavioral and Biochemical Alteration in Male Albino Mice. Pharmacogn. Res..

[12] Fernandes C.P., de Almeida F.B., Silveira A.N., Gonzalez M.S., Mello C.B., Feder D., Apolinário R., Santos M.G., Carvalho J.C.T., Tietbohl L.A.C. (2014). Development of an Insecticidal Nanoemulsion with Manilkara Subsericea (Sapotaceae) Extract. J. Nanobiotechnol..

[13] Tsai Y.-J., Chen B.-H. (2016). Preparation of Catechin Extracts and Nanoemulsions from Green Tea Leaf Waste and Their Inhibition Effect on Prostate Cancer Cell PC-3. Int. J. Nanomed..

[14] Li F., Song S., Guo Y., Zhao Q., Zhang X., Pan W., Yang X. (2015). Preparation and Pharmacokinetics Evaluation of Oral Self-Emulsifying System for Poorly Water-Soluble Drug Lornoxicam. Drug Deliv..

[15] Morsi N., Ibrahim M., Refai H., El Sorogy H. (2017). Nanoemulsion-Based Electrolyte Triggered in Situ Gel for Ocular Delivery of Acetazolamide. Eur. J. Pharm. Sci..

[16] Khalil H.M.A., Mahmoud D.B., El-Shiekh R.A., Bakr A.F., Boseila A.A., Mehanna S., Naggar R.A., Eliwa H.A. (2022). Antidepressant and Cardioprotective Effects of Self-Nanoemulsifying Self-Nanosuspension Loaded with *Hypericum perforatum* on Post-Myocardial Infarction Depression in Rats. AAPS PharmSciTech.

[17] Baghcheghi Y., Hosseini M., Beheshti F., Salmani H., Anaeigoudari A. (2018). Thymoquinone Reverses Learning and Memory Impairments and Brain Tissue Oxidative Damage in Hypothyroid Juvenile Rats. Arq. Neuro-Psiquiatr..

[18] Elbardisy B., Galal S., Abdelmonsif D.A., Boraie N. (2019). Intranasal Tadalafil Nanoemulsions: Formulation, Characterization and Pharmacodynamic Evaluation. Pharm. Dev. Technol..

[19] Owoeye O., Adedara I.A., Farombi E.O. (2018). Pretreatment with Taurine Prevented Brain Injury and Exploratory Behaviour Associated with Administration of Anticancer Drug Cisplatin in Rats. Biomed. Pharmacother..

[20] Ridzuan N.A., Teoh S.L., Rashid N.A., Othman F., Baharum S.N., Hussan F. (2019). Polygonum Minus Ethanolic Extracts Attenuate Cisplatin–Induced Oxidative Stress in the Cerebral Cortex of Rats via Its Antioxidant Properties. Asian Pac. J. Trop. Biomed..

[21] Khalil H.M.A., Azouz R.A., Hozyen H.F., Aljuaydi S.H., AbuBakr H.O., Emam S.R., Al-Mokaddem A.K. (2022). Selenium Nanoparticles Impart Robust Neuroprotection against Deltamethrin-Induced Neurotoxicity in Male Rats by Reversing Behavioral Alterations, Oxidative Damage, Apoptosis, and Neuronal Loss. Neurotoxicology.

[22] Khalil H.M.A., Khalil I.A., Al-Mokaddem A.K., Hassan M., El-Shiekh R.A., Eliwa H.A., Tawfek A.M., El-Maadawy W.H. (2022). Ashwagandha-Loaded Nanocapsules Improved the Behavioral Alterations, and Blocked MAPK and Induced Nrf2 Signaling Pathways in a Hepatic Encephalopathy Rat Model. Drug Deliv. Transl. Res..

[23] Livak K.J., Schmittgen T.D. (2001). Analysis of Relative Gene Expression Data Using Real-Time Quantitative PCR and the 2(-Delta Delta C(T)) Method. Methods.

[24] Bancroft J.D. (2008). Theory and Practice of Histological Techniques.

[25] Ali K.A., El-Naa M.M., Bakr A.F., Mahmoud M.Y., Abdelgawad E.M., Matoock M.Y. (2022). The Dual Gastro- and Neuroprotective Effects of Curcumin Loaded Chitosan Nanoparticles against Cold Restraint Stress in Rats. Biomed. Pharmacother..

[26] Elghazawy N.H., Zaafar D., Hassan R.R., Mahmoud M.Y., Bedda L., Bakr A.F., Arafa R.K. (2022). Discovery of New 1,3,4-Oxadiazoles with Dual Activity Targeting the Cholinergic Pathway as Effective Anti-Alzheimer Agents. ACS Chem. Neurosci..

[27] Meirelles G., Bridi H., Stolz E.D., Teixeira H.F., von Poser G.L., Rates S.M.K. (2017). Nanoemulsion Improves Antinociceptive Activity of HP1, a Benzopyran from Hypericum Polyanthemum. Planta Med. Int. Open.

[28] Kandeil M.A., Gomaa S.B., Mahmoud M.O. (2020). The Effect of Some Natural Antioxidants against Cisplatin-Induced Neurotoxicity in Rats: Behavioral Testing. Heliyon.

[29] Ongnok B., Chattipakorn N., Chattipakorn S.C. (2020). Doxorubicin and Cisplatin Induced Cognitive Impairment: The Possible Mechanisms and Interventions. Exp. Neurol..

[30] Lomeli N., Di K., Czerniawski J., Guzowski J.F., Bota D.A. (2017). Cisplatin-Induced Mitochondrial Dysfunction Is Associated with Impaired Cognitive Function in Rats. Free. Radic. Biol. Med..

[31] Kraeuter A.-K., Guest P.C., Sarnyai Z., Guest P.C. (2019). The Y-Maze for Assessment of Spatial Working and Reference Memory in Mice. Pre-Clinical Models: Techniques and Protocols.

[32] Bezu M., Maliković J., Kristofova M., Engidawork E., Höger H., Lubec G., Korz V. (2017). Spatial Working Memory in Male Rats: Pre-Experience and Task Dependent Roles of Dopamine D1- and D2-Like Receptors. Front. Behav. Neurosci..

[33] Almutairi M.M., Alanazi W.A., Alshammari M.A., Alotaibi M.R., Alhoshani A.R., Al-Rejaie S.S., Hafez M.M., Al-Shabanah O.A. (2017). Neuro-Protective Effect of Rutin against Cisplatin-Induced Neurotoxic Rat Model. BMC Complement. Altern. Med..

[34] Deponte M. (2013). Glutathione Catalysis and the Reaction Mechanisms of Glutathione-Dependent Enzymes. Biochim. Biophys. Acta (BBA)-Gen. Subj..

[35] Turan M., Cayir A., Cetin N., Suleyman H., Turan I.S., Tan H. (2014). An Investigation of the Effect of Thiamine Pyrophosphate on Cisplatin-Induced Oxidative Stress and DNA Damage in Rat Brain Tissue Compared with Thiamine: Thiamine and Thiamine Pyrophosphate Effects on Cisplatin Neurotoxicity. Hum. Exp. Toxicol..

[36] Xie A., Gao J., Xu L., Meng D. (2014). Shared Mechanisms of Neurodegeneration in Alzheimer’s Disease and Parkinson’s Disease. BioMed Res. Int..

[37] Gupta P., Makkar T.K., Goel L., Pahuja M. (2022). Role of Inflammation and Oxidative Stress in Chemotherapy-Induced Neurotoxicity. Immunol. Res..

[38] Domingo I.K., Latif A., Bhavsar A.P. (2022). Pro-Inflammatory Signalling PRRopels Cisplatin-Induced Toxicity. Int. J. Mol. Sci..

[39] Prabhakaran J., Molotkov A., Mintz A., Mann J.J. (2021). Progress in PET Imaging of Neuroinflammation Targeting COX-2 Enzyme. Molecules.

[40] Liu X., Yao C., Tang Y., Liu X., Duan C., Wang C., Han F., Xiang Y., Wu L., Li Y. (2022). Role of P53 Methylation in Manganese-Induced Cyclooxygenase-2 Expression in BV2 Microglial Cells. Ecotoxicol. Environ. Saf..

[41] Manohar S., Jamesdaniel S., Salvi R. (2014). Cisplatin Inhibits Hippocampal Cell Proliferation and Alters the Expression of Apoptotic Genes. Neurotox Res..

[42] Bisht A., Dickens M., Rutherfurd-Markwick K., Thota R., Mutukumira A.N., Singh H. (2020). Chlorogenic Acid Potentiates the Anti-Inflammatory Activity of Curcumin in LPS-Stimulated THP-1 Cells. Nutrients.

[43] Girsang E., Lister I.N.E., Ginting C.N., Nasution S.L., Suhartina S., Munshy U.Z., Rizal R., Widowati W. (2019). Antioxidant and Anti-Inflammatory Activity of Chlorogenic Acid on Lead-Induced Fibroblast Cells. J. Phys. Conf. Ser..

[44] Maslin L.A., Weeks B.R., Carroll R.J., Byrne D.H., Turner N.D. (2022). Chlorogenic Acid and Quercetin in a Diet with Fermentable Fiber Influence Multiple Processes Involved in DSS-Induced Ulcerative Colitis but Do Not Reduce Injury. Nutrients.

[45] Abdel Motaal A., Ezzat S.M., Tadros M.G., El-Askary H.I. (2016). In Vivo Anti-Inflammatory Activity of Caffeoylquinic Acid Derivatives from *Solidago Virgaurea* in Rats. Pharm. Biol..

[46] Soquetta M.B., de Terra L.M., Bastos C.P. (2018). Green Technologies for the Extraction of Bioactive Compounds in Fruits and Vegetables. CyTA J. Food.

[47] Nakanishi T., Mukai K., Yumoto H., Hirao K., Hosokawa Y., Matsuo T. (2010). Anti-Inflammatory Effect of Catechin on Cultured Human Dental Pulp Cells Affected by Bacteria-Derived Factors. Eur. J. Oral Sci..

[48] Gomez del Rio M.A., Sanchez-Reus M.I., Iglesias I., Pozo M.A., Garcia-Arencibia M., Fernandez-Ruiz J., Garcia-Garcia L., Delgado M., Benedi J. (2013). Neuroprotective Properties of Standardized Extracts of *Hypericum perforatum* on Rotenone Model of Parkinson’s Disease. CNS Neurol. Disord.-Drug Targets-CNS Neurol. Disord..

[49] Sharifi-Rad J., Rodrigues C.F., Sharopov F., Docea A.O., Can Karaca A., Sharifi-Rad M., Kahveci Karıncaoglu D., Gülseren G., Şenol E., Demircan E. (2020). Diet, Lifestyle and Cardiovascular Diseases: Linking Pathophysiology to Cardioprotective Effects of Natural Bioactive Compounds. Int. J. Environ. Res. Public Health.

[50] Farcas A.D., Mot A.C., Zagrean-Tuza C., Ticolea M., Sevastre B., Kulak M., Silaghi-Dumitrescu R., Parvu A. (2019). Remarkable Rutin-Rich Hypericum Capitatum Extract Exhibits Anti-Inflammatory Effects on Turpentine Oil-Induced Inflammation in Rats. BMC Complement. Altern. Med..

[51] Fuda H., Watanabe M., Hui S.-P., Joko S., Okabe H., Jin S., Takeda S., Miki E., Watanabe T., Chiba H. (2015). Anti-Apoptotic Effects of Novel Phenolic Antioxidant Isolated from the Pacific Oyster (Crassostrea Gigas) on Cultured Human Hepatocytes under Oxidative Stress. Food Chem..

[52] Diao J., Ou J., Dai H., Li H., Huang W., Hua H., Xie T., Wang M., Yang Y. (2020). Antioxidant and Antiapoptotic Polyphenols from Green Tea Extract Ameliorate CCl4-Induced Acute Liver Injury in Mice. Chin. J. Integr. Med..

[53] Foroutanfar A., Mehri S., Kamyar M., Tandisehpanah Z., Hosseinzadeh H. (2020). Protective Effect of Punicalagin, the Main Polyphenol Compound of Pomegranate, against Acrylamide-Induced Neurotoxicity and Hepatotoxicity in Rats. Phytother. Res..

[54] Zirak N., Shafiee M., Soltani G., Mirzaei M., Sahebkar A. (2019). *Hypericum perforatum* in the Treatment of Psychiatric and Neurodegenerative Disorders: Current Evidence and Potential Mechanisms of Action. J. Cell. Physiol..

[55] Javadinia S.S., Abbaszadeh-Goudarzi K., Mahdian D., Hosseini A., Ghalenovi M., Javan R. (2022). A Review of the Protective Effects of Quercetin-Rich Natural Compounds for Treating Ischemia-Reperfusion Injury. Biotech. Histochem..

[56] Wu P.-S., Ding H.-Y., Yen J.-H., Chen S.-F., Lee K.-H., Wu M.-J. (2018). Anti-Inflammatory Activity of 8-Hydroxydaidzein in LPS-Stimulated BV2 Microglial Cells via Activation of Nrf2-Antioxidant and Attenuation of Akt/NF-ΚB-Inflammatory Signaling Pathways, as well as Inhibition of COX-2 Activity. J. Agric. Food Chem..

[57] Cui L., Xu F., Wu K., Li L., Qiao T., Li Z., Chen T., Sun C. (2020). Anticancer Effects and Possible Mechanisms of Lycopene Intervention on N-Methylbenzylnitrosamine Induced Esophageal Cancer in F344 Rats Based on PPARγ1. Eur. J. Pharmacol..

[58] Dost T., Ozkayran H., Gokalp F., Yenisey C., Birincioglu M. (2009). The Effect of *Hypericum perforatum* (St. John’s Wort) on Experimental Colitis in Rat. Dig. Dis. Sci..

[59] Novelli M., Masiello P., Beffy P., Menegazzi M. (2020). Protective Role of St. John’s Wort and Its Components Hyperforin and Hypericin against Diabetes through Inhibition of Inflammatory Signaling: Evidence from In Vitro and In Vivo Studies. Int. J. Mol. Sci..

[60] Yang Z., Cai X., Xu A., Xu F., Liang Q. (2015). Bone Marrow Stromal Cell Transplantation through Tail Vein Injection Promotes Angiogenesis and Vascular Endothelial Growth Factor Expression in Cerebral Infarct Area in Rats. Cytotherapy.

[61] Sherif I.O., Al-Shaalan N.H., Sabry D. (2021). Neuroprotective Potential of Bone Marrow-Derived Mesenchymal Stem Cells Following Chemotherapy. Biomedicines.

