# Correction: Khalil et al. *Hypericum perforatum* L. Nanoemulsion Mitigates Cisplatin-Induced Chemobrain via Reducing Neurobehavioral Alterations, Oxidative Stress, Neuroinflammation, and Apoptosis in Adult Rats. *Toxics* 2023, *11*, 159

**DOI:** 10.3390/toxics13060437

**Published:** 2025-05-26

**Authors:** Heba M. A. Khalil, Hanan M. A. El Henafy, Islam A. Khalil, Alaa F. Bakr, Mohamed I. Fahmy, Nancy S. Younis, Riham A. El-Shiekh

**Affiliations:** 1 Department of Veterinary Hygiene and Management, Faculty of Veterinary Medicine, Cairo University, Giza 12211, Egypt; 2 Medical Laboratory Department, Faculty of Applied Medical Sciences, October 6 University, Giza 3230911, Egypt; 3 Department of Pharmaceutics, College of Pharmaceutical Sciences and Drug Manufacturing, Misr University of Science and Technology (MUST), Giza 12582, Egypt; 4 Department of Pathology, Faculty of Veterinary Medicine, Cairo University, Giza 12211, Egypt; 5 Department of Pharmacology and Toxicology, Faculty of Pharmacy, Heliopolis University, Cairo 2834, Egypt; 6 Department of Pharmaceutical Sciences, College of Clinical Pharmacy, King Faisal University, Al-Ahsa 31982, Saudi Arabia; 7 Department of Pharmacognosy, Faculty of Pharmacy, Cairo University, Kasr el Aini St., Cairo 11562, Egypt

## Error in Figure

In the original publication [1], there was a mistake in Figure 7 (DG–control group) as published. We identified an unintended mixing up during the preparation of the figures. The corrected Figure 7 appears below. The authors state that the scientific conclusions are unaffected. This correction was approved by the Academic Editor. The original publication has also been updated.

## Figures and Tables

**Figure 7:**
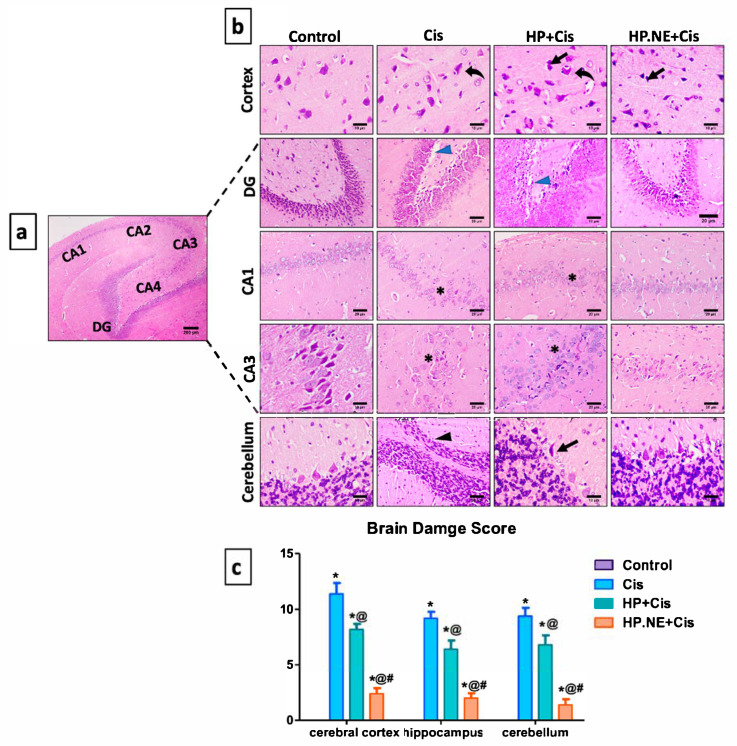
Effect of HP L. and HP.NE administration on the histological structure of the brain. (**a**) Photomicrograph from control group presenting the normal structure of the hippocampal area, which is made up of Cornu Ammonis (CA1, CA2, CA3, and CA4) and Dentate gyrus (DG); scale bar denotes 200 m. (**b**) Photomicrograph of brain tissues stained with H&E; scale bars denote 10 and 20 m. Notable symbols on the figure point to neurophagia of degenerated neurons (curved arrows), dark stained neurons (arrows), loss of neurons (black arrowheads), areas void of neurons (*), and vacuolation (blue arrowheads). (**c**) Brain damage score. Results are expressed as median standard deviation (SD), determined using the Kruskal–Wallis test followed by Dunn’s multiple comparison test. * means significant from the control group; @, significant from the Cis group; #, significant from HP + Cis group.
