# Supplementary material for: Hypericum perforatum L. Nanoemulsion Mitigates Cisplatin-Induced Chemobrain via Reducing Neurobehavioral Alterations, Oxidative Stress, Neuroinflammation, and Apoptosis in Adult Rats
Source: Toxics. 2023 Feb 8;11(2):159. doi: 10.3390/toxics11020159 (PMC9961500; doi:10.3390/toxics11020159)
Supplement: Supplementary file 1 [file toxics-11-00159-s001.zip › toxics-2179091-supplementary.pdf]

Hypericum perforatum L. nanoemulsion mitigates cisplatin-induced chemobrain via reducing neurobehavioral alterations, oxidative stress, neuroinflammation, and apoptosis in adult rats

Heba M. A. Khalil[1*], Hanan M. A. El Henafy[2], Islam A. Khalil[3], Alaa F. Bakr[4], Mohamed I. Fahmy[5], Nancy S. Younis[6], Riham A. El-Shiekh[7]

1 Department of Veterinary Hygiene and Management, Faculty of Veterinary Medicine, Cairo University, Giza 12211, Egypt

2 Medical Laboratory Department, Faculty of Applied Medical Sciences, October 6 University, Giza 3230911, Egypt

3 Department of Pharmaceutics, College of Pharmaceutical Sciences and Drug Manufacturing, Misr University of Science and Technology (MUST), 6th of October, Giza 12582, Egypt

4 Department of pathology, Faculty of Vet. Medicine, Cairo University, Giza, 12211, Egypt

5 Department of pharmacology and Toxicology, Heliopolis University, Cairo 2834, Egypt

6 Department of Pharmaceutical Sciences, College of Clinical Pharmacy, King Faisal University, Al-Ahsa 31982, Saudi Arabia

7 Department of Pharmacognosy, Faculty of Pharmacy, Cairo University, Kasr el Aini st., Cairo 11562, Egypt

*Correspondence: heba.ali315@gmail.com; heba.ali@cu.edu.eg; Tel.: +201013666331

Supplementary Material

List of contents

No	Content	Page
**Figure S1**	Total ion chromatogram of HP L. ethanolic extract in positive ionization mode.	S2
**Table S1**	Metabolites identified from the ethanolic extract of HP L.	S3

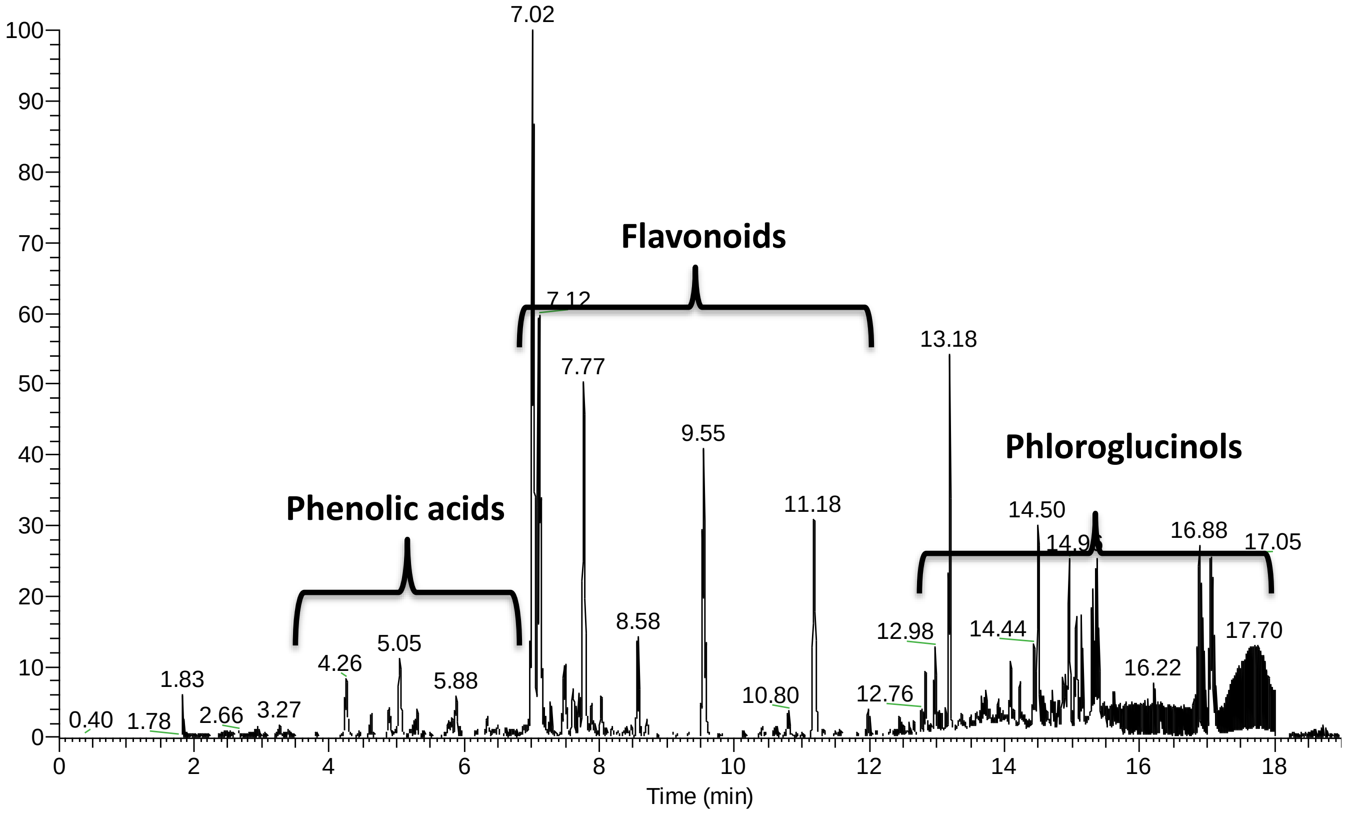

Figure S1. Total ion chromatogram of HP L. ethanolic extract in positive ionization mode.

Table S1. Metabolites identified from the ethanolic extract of HP L.

R_t	MS^+	Error (ppm)	Identified compound	Molecular formula	MS/MS^+ fragments
4.26	355.1026	0.68	5-*O*-caffeoylquinic acid (chlorogenic acid)	$C_{16}H_{19}O_9$	163.03, 135.044
5.03	355.1025	0.511	*O*-caffeoylquinic acid isomer	$C_{16}H_{19}O_9$	291.08, 163.03
5.29	579.15	0.444	Procyanidin dimer	$C_{30}H_{27}O_{12}$	485.16, 289.07,195.06, 177.05
5.76	291.0864	0.499	Catechin	$C_{15}H_{15}O_6$	234.98
6.42	481.0984	1.44	Myricetin 3-*O*-glucoside	$C_{21}H_{21}O_{13}$	319.04, 137.023
6.72	611.1611	0.669	Rutin	$C_{27}H_{31}O_{16}$	303.04, 207.13
6.88	611.1616	1.471	Kaempferol-*O*-glucopyranosyl-galactopyranoside	$C_{27}H_{31}O_{16}$	287.05, 153.01
7.08	479.0825	0.925	Miquelianin	$C_{21}H_{19}O_{13}$	303.05
7.14	465.1033	1.113	Quercetin-3-*O*-galactoside (hyperoside)	$C_{21}H_{21}O_{12}$	303.05
7.27	451.1242	1.534	Dihydroquercetrin (astilbin)	$C_{21}H_{23}O_{11}$	303.049
7.49	435.0927	1.269	Quercetin 3-*O*-arabinopyranside	$C_{20}H_{19}O_{11}$	303.04, 149.02
7.5	449.1085	1.519	Quercetin-3-*O*-rhamnoside (isoquercetrin)	$C_{21}H_{21}O_{11}$	303.04, 153.01
7.61	435.0927	1.2	Quercetin 3-*O*-arabinopyranside	$C_{20}H_{19}O_{11}$	303.05
7.9	433.1136	1.609	Vitexin	$C_{21}H_{21}O_{10}$	271.05, 154.99
9.47	701.15027	0.247	Skyrin-2-O-glucopyranoside	$C_{36}H_{29}O_{15}$	539.09
9.55	303.05	0.102	Quercetin	$C_{15}H_{11}O_7$	229.04, 153.01
10.8	287.0548	-0.713	Kaempferol	$C_{15}H_{11}O_6$	153.018
11.16	539.0979	1.163	Amentaflavone (Biapigenin)	$C_{30}H_{19}O_{10}$	445.25, 293.13
13.34	521.0864	-0.534	Pseudohypericin	$C_{30}H_{17}O_9$	471.31, 293.13
14.75	401	-5.672	Hyperatomanin/hypercalyxone A	$C_{25}H_{37}O_4$	293.13
15.17	569.38416	0.868	Hydroperoxy furohyperforin	$C_{35}H_{53}O_6$	493.31
16.22	553.3897	1.624	Furohyperforin	$C_{35}H_{53}O_5$	485.32, 293.137
16.32	553.3897	1.407	Furohyperforin	$C_{35}H_{53}O_5$	293.13
16.26	483.3473	0.897	Hyperpolyphyllirin	$C_{31}H_{47}O_4$	293.13
16.66	469.3315	0.519	Hyperfirin	$C_{30}H_{45}O_4$	287.16
16.67	537.3948	1.756	Hyperforin	$C_{35}H_{53}O_4$	469.33, 277.14
16.91	551.4094	-0.23	Adhyperforin	$C_{36}H_{55}O_4$	293.13
